# Underlying anti-cancer mechanisms of histone deacetylase (HDAC) inhibitors in tamoxifen-resistant breast cancer cells

**DOI:** 10.22038/IJBMS.2024.76157.16478

**Published:** 2024

**Authors:** Lingyan Wang, Yukai Xu, Chunhui Gao

**Affiliations:** 1 Department of Breast Surgery, Baoding First Central Hospital, Baoding, Hebei, 071000, China; 2 Chengde Medical College, Chengde, Hebei, China

**Keywords:** Anti-cancer, Breast cancer, Drug resistance, Histone deacetylase- inhibitors, Tamoxifen

## Abstract

**Objective(s):**

Breast cancer is an important women’s malignancy with high cancer-related deaths worldwide. Drug resistance lowers the treatment efficacy in this malignancy. This study aimed to explore the underlying mechanisms of histone deacetylase (HDAC) inhibitor trichostatin A (TSA) to overcome resistance to tamoxifen in breast cancer cells.

**Materials and Methods:**

Tamoxifen-resistance in MCF-7 breast cancer cells was simulated. MTT assay was used to detect the cytotoxic effects of HDAC inhibitor and PI3K inhibitor on the cancer cells. Trans-well assay was applied to evaluate the invasion and migration of the treated cancer cells. Flow cytometer assay was also applied to evaluate cell cycle phases in the treated cancer cells. Finally, expression of vascular endothelial growth factor (VEGF), E-cadherin, Vimentin, phosphorylated phosphatidylinositol kinase (p-PI3k), phosphorylated protein kinase B (p-AKT), and phosphorylated mammalian target protein of rapamycin (p-mTOR) was evaluated by western blotting.

**Results:**

The obtained results indicated that HDAC inhibitor treatments significantly decreased viability, migration, and invasion in the cancer cells. Furthermore, the frequency of the treated cancer cells significantly increased in the S phase as well as significantly decreasing in the G2/M phase of the cell cycle. Moreover, HDAC inhibitor modified levels of VEGF, E-cadherin, Vimentin, p-PI3k, p-AKT, and p-mTOR proteins. However, HDAC inhibitor combined with PI3K inhibitor exerts more profound effects on the cancer cells as compared to HDAC inhibitor monotherapy.

**Conclusion:**

HDAC inhibitors inhibited the survival of breast cancer drug-resistant cells, invasion, migration, and angiogenesis by inhibiting the PI3k/Akt/mTOR signaling pathway.

## Introduction

Breast cancer is one of the most common malignant tumors in clinical practice, especially in women. It is only the second malignant tumor that causes female deaths after lung cancer. Relevant statistics showed that more than 65% of breast cancer patients are positive for estrogen receptor expression. The incidence of breast cancer is increasing year by year and gradually younger with the development of the economy, increasing social pressure, and changes in people’s lifestyles (1, 2). Currently, the clinical treatment of hormone-dependent breast cancer mainly uses endocrine therapy, which can significantly delay the progression of this disease. However, endocrine therapy resistance is the leading cause of treatment failure in patients (2). 

Tamoxifen is a commonly used drug in clinical endocrine therapy, but long-term use can cause drug resistance in patients. The therapeutic efficacy of tamoxifen is significantly decreased in drug‐resistant cancer cells (3). Therefore, development of different therapeutic methods such as application of chemotherapeutic agents or combination regimens was necessary for the treatment and prolonged survival of patients with breast cancer (4).

Histone deacetylase (HDAC) inhibitor is a new anti-tumor drug that can induce tumor cell apoptosis and differentiation by activating the expression of tumor suppressor genes without an obvious effect on normal cells (5). Studies have found that the mechanism by which HDAC inhibitors can reverse tumor drug resistance to a certain extent is not clear. HDACs have been illustrated to play a key role corresponding to proliferation, migration, and invasion in different types of cancer. HDACs are an enzyme family, and their activity rules the lysine residues of proteins in the acetylation state, notably those present in the amino-terminal extensions of the core histones. The expression of a gene is exaggerated by acetylation of histones, as is its impact on chromatin configuration; the most important role of this enzyme is controlling the cell cycle, cell survival, cell progression, and differentiation (6, 7). Currently, several HDAC inhibitors have been approved by the FDA for the treatment of hematological malignancies, including vorinostat (SAHA), belinostat (PXD-101), romidepsin (FK-228), and Panobinostat (LBH589). However, HDAC inhibitors have shown limited success in the treatment of solid tumors (8, 9).

There is only a limited number of studies reporting the effects of HDAC inhibitors in tamoxifen-resistant cancer cells. However, there is no study on anti-cancer activity and associated mechanisms of combined HDAC inhibitor and PI3K inhibitor against breast cancer cells. In the present study, we investigated the *in vitro* inhibitory effects of combination therapy with HDAC inhibitor and PI3K inhibitor against breast cancer cell line (MCF-7), as well as associated mechanisms to provide the scientific rationale for clinical application in the treatment of breast cancer.

## Materials and Methods

***Cancer cell culture***

The breast cell line MCF-7 was purchased (Shanghai Fuheng Biotechnology Co., Ltd.) and cultured in DMEM containing 10% fetal bovine serum and penicillin-streptomycin double-antibody solution (100 IU/ml). The cancer cells then were incubated in 95% humidity, 5% CO_2_, and 37 ^°^C conditions. Cells were subcultured when the cell density reached 75%~85%.

***Preparation of tamoxifen-resistant cancer cell***

Construction of MCF-7 tamoxifen-resistant (MCF-7/TAMR) cell line was performed in the logarithmic growth phase of the cancer cells cultured in DMEM medium supplemented with 0.2 μM tamoxifen. The cells were cultured at 85% density in a culture medium containing a high concentration of tamoxifen until MCF-7/TAMR cells became tolerant to 2 μM tamoxifen. In order to maintain drug resistance, the MCF-7/TAMR drug-resistant cell line was switched to a culture medium containing 0.2 μM tamoxifen.

***Cell viability assay***

MTT assay was used to detect the effect of different concentrations of HDAC inhibitor and PI3K inhibitor on the viability of MCF-7/TAMR cells. The cancer cells were seeded into 96-well plates (10000 cells/well) and incubated for 24 hr. The cells were treated with different concentrations of HDAC inhibitor (200 nmol/L Trichostatin A, 10 μM) and HDAC inhibitor+PI3K inhibitor (200 nmol/l Trichostatin A+10 μmol/L PI3K inhibitor LY294002, 10 μM) for 48 hr. MTT solution (5 mg/ml, 10 μl) was respectively added to each well at 0, 12, 24, and 48 hr, and the absorbance value at 520 nm of each group of cells was measured with a microplate reader (Bio-Rad, United States of America). 

***Cell cycle analysis***

The cell cycle phase of the treated cancer cells was evaluated by the PI staining flow cytometry method. The cancer cells were seeded in a 6-well plate (1.5×10^5^ cells/well) treated with different concentrations of HDAC inhibitor and PI3K inhibitor, and incubated for 48 hr. The cells were stained by PI solution (50 μg/ml) and incubated for 1 hr. Different cell cycle phases in the cancer cells were analyzed by a flow cytometry instrument (Miltenyi Biotec, Germany).

***Trans-well migration assay***

Trans-well assay was used to measure the changes in cell invasion and migration ability of treated cancer cells. The cancer cells were seeded in the upper chamber containing 200 μl of serum-free medium and inhibitors, also 400 μl of complete medium containing 15% fetal bovine serum was added in the lower chamber. After 24 hr, non-migrated cells in the upper chamber were removed, and the migrated cells in the bottom chamber were stained with crystal violet. Finally, the invaded and migrated cancer cells were counted in five random fields using an inverted phase‐contrast microscope (Olympus, Japan).

***Western blot analysis***

Western blotting was used to detect the protein expression level of vascular endothelial growth factor (VEGF), E-cadherin, Vimentin, phosphorylated phosphatidylinositol kinase (p-PI3k), phosphorylated protein kinase B (p-AKT), and phosphorylated mammalian target protein of rapamycin (p-mTOR). Total protein was extracted from cultured MCF-7/TAMR cells using a total protein extraction kit and quantified using a BCA protein assay kit. The extracted protein samples were separated by sodium dodecyl sulfate-polyacrylamide gel electrophoresis (SDS-PAGE) and transferred to a polyvinylidene difluoride (PVDF) membrane. The membrane was blocked by dry nonfat milk (4%) and primary antibody (1:1000) was added at 4 ^°^C for 24 hr. The horseradish peroxidase (HRP) conjugated rabbit antibody (1:5000) was added as a secondary antibody at room temperature for 60 min. The western blot was developed on X-ray films and the optical density (OD) of the protein bands was measured by ImageJ software. The protein bands were normalized to β-actin protein.

***Statistical analysis***

All experiments were repeated three times. The obtained data are shown as the mean±standard error and analyzed by SPSS 19.0 statistics software. The SNK-q test was used between the two groups, and the single-factor multi-sample mean comparison was used between multiple groups. *P*<0.05 was considered statistically significant. 

## Results

***Cancer cell viability***

Treatment with HDAC inhibitor significantly decreased the viability of the breast cancer cells in a time- and dose-dependent manner (*P*<0.05). Particularly, after treatment, the survival rate of the cancer cells decreased by 49.48% and 60.78% in 24 and 48 hr, respectively (Table 1).

***Cancer cell cycle***

Compared with the control group, the proportion of S-phase in the cancer cells treated by HDAC inhibitor was significantly increased; while the ratio of G2/M phase was significantly reduced (*P*<0.05). In comparison with the HDAC inhibitor treatment, the S phase ratio in the cancer cells treated by HDAC inhibitor+PI3K inhibitor was significantly decreased, and the G2/M ratio phase was significantly increased (*P*<0.05). The results indicated that HDAC inhibitor+PI3K inhibitor combination therapy exerts more profound effects on the cell cycle change as compared to HDAC inhibitor or PI3K inhibitor monotherapy (Table 2).

***Cancer cell migration***

**Figure 1:**
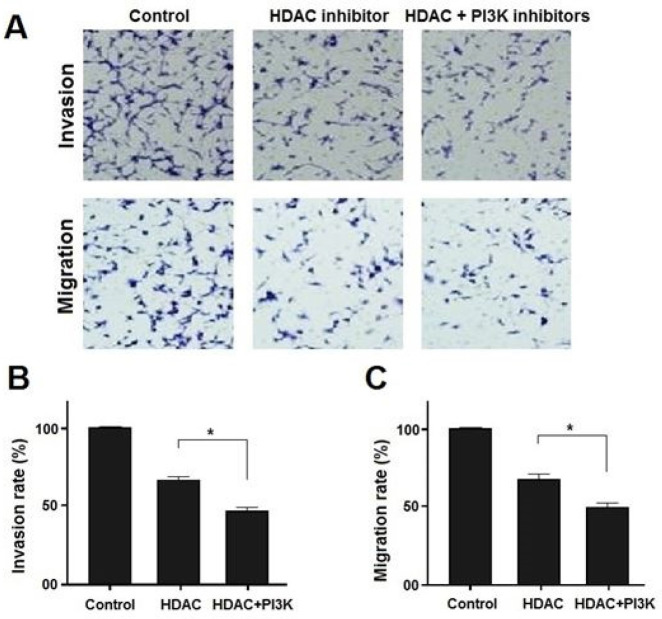
Invasion and migration rate of the treated cancer cells by histone deacetylase inhibitors for 24 hr (magnification 200×)

Compared with the drug-resistant group, the number of migrated cancer cells treated by HDAC inhibitor significantly increased (*P*<0.05). Moreover, the number of migrated cancer cells treated by HDAC inhibitor+PI3K inhibitor was significantly reduced as compared with the HDAC inhibitor (*P*<0.05). The results demonstrated that HDAC inhibitor+PI3K inhibitor combination therapy exerts more profound effects on the inhibition of cancer cell migration and invasion as compared to HDAC inhibitor or PI3K inhibitor alone (Table 3). The cancer cell migration in the treatment groups is presented in Figure 1.

***Cancer-related proteins expression***

**Figure 2:**
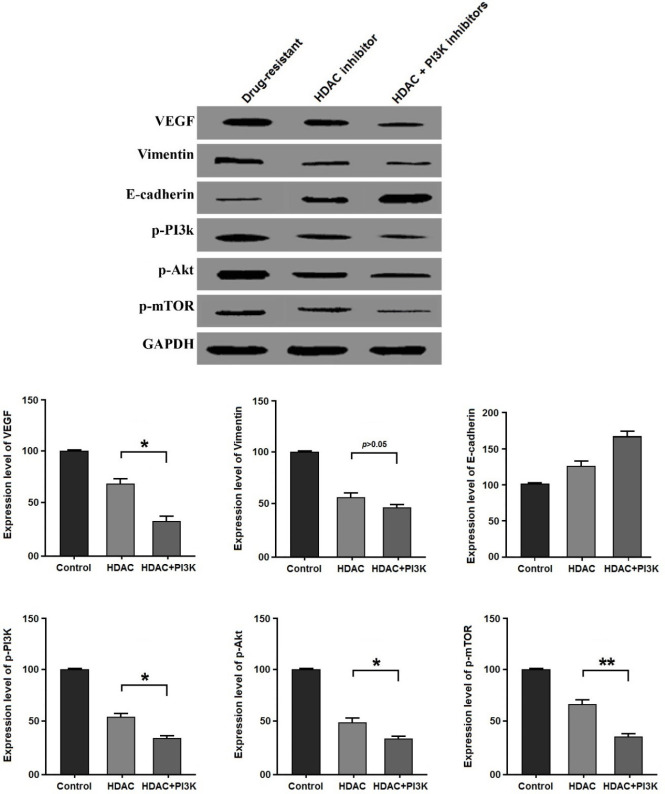
Expression levels of cancer-related proteins in the treated cancer cells by histone deacetylase inhibitors for 24 hr

The protein expression level of VEGF, E-cadherin, Vimentin, p-PI3k, p-Akt, and p-mTOR in the cancer cells treated by HDAC inhibitor was significantly reduced compared with the drug-resistant group, along with significantly increased expression level of E-cadherin (*P*<0.05). In comparison with the HDAC inhibitor treatment, VEGF, Vimentin, p-PI3k, p-Akt, and p-mTOR protein expression levels were significantly decreased in the cancer cells treated by HDAC inhibitor+PI3K inhibitor; while E-cadherin protein level was significantly increased (*P*<0.05). The target protein levels in the treated groups are presented in Figure 2.

**Table 1:** Survival rate of the treated cancer cells by histone deacetylase inhibitors

**Concentration HDAC inhibitors**	**Cell survival rate (%)**			
	**0 hr**	**12 hr**	**24 hr**	**48 hr**
0 nmol/L	100.00±0.01	92.51±2.18	88.29±2.31	86.19±2.81
50 nmol/L	93.83±1.76^a^	74.78±3.58^a^	70.54±2.16^a^	57.17±1.46^a^
100 nmol/L	87.88±2.35^ab^	68.53±3.74^ab^	62.18±1.24^ab^	51.79±3.17^ab^
200 nmol/L	80.28±1.14^abc^	62.18±2.41^abc^	50.52±2.38^abc^	39.22±2.29^abc^
p-value	<0.001	<0.001	<0.001	<0.001

Values are shown as the mean±standard error (n=4). ^a ^*P<*0.05: compared with 0 nmol/l group; ^b ^*P<*0.05: compared with 50 nmol/l group; ^c ^*P<*0.05: compared with 100 nmol/l group

HDAC: histone deacetylase

**Table 2:** Apoptosis rate of the treated cancer cells by histone deacetylase inhibitors

**Groups**	**Cell cycle phases (%)**		
	**G0/G1**	**S**	**G2/M **
Drug resistant	45.55±1.68	36.78±2.13	16.48±2.12
HDAC inhibitor	49.33±3.24	58.74±2.87^a^	2.69±1.58^a^
HDAC + PI3K inhibitors	47.17±2.09	42.16±2.18^b^	9.57±1.64^b^
p-value	0.142	<0.001	<0.001

Values are shown as the mean±standard error (n=4). ^a ^*P<*0.05: compared with drug resistance group: ^b ^*P<*0.05; compared with HDAC inhibitor group

HDAC: histone deacetylase

**Table 3:** Invasion and migration rate of the treated cancer cells by histone deacetylase inhibitors

**Groups**	**Invaded cells**	**Migrated cells**
Drug-resistant	130.58±6.17	156.22±12.69
HDAC inhibitor	70.35±4.18^a^	80.65±2.41^a^
HDAC + PI3K inhibitor	81.68±4.66^b^	101.26±7.84^b^
p-value	<0.001	<0.001

Values are shown as the mean±standard error (n=4). ^a^ *P<*0.05: compared with drug resistance group: ^b^ *P<*0.05; compared with HDAC inhibitor group

HDAC: histone deacetylase

(A) drug-resistant group; (B) HDAC inhibitor group; (C) HDAC inhibitor+PI3K inhibitor group (*P<0.05); HDAC: histone deacetylase

## Discussion

Breast cancer is one of the most common malignant tumors in women worldwide. It is a complex heterogeneous tumor with various unique pathological features and biological behavior entities (10). Understanding of the molecular mechanisms of breast cancer occurrence, development, and metastasis has also been deepened with the continuous development of molecular biology theory and technology (11, 12). Estrogen receptor alpha plays a vital role in developing breast cancer as a member of the estrogen model pathway. Patients with estrogen receptor-positive breast cancer account for most of breast cancer patients, while patients with estrogen receptor-positive breast cancer have a poor prognosis and are prone to tamoxifen resistance (13, 14). Therefore, overcoming tamoxifen resistance and improving the sensitivity of patients to chemotherapy have an essential role in improving the treatment effect and prognosis of patients. The balance of histone deacetylation status has an important relationship with the occurrence and development of various malignant tumors due to the specificity of the human HDAC gene location (15).

Cell proliferation activity and apoptosis are important characteristics of malignant tumors, while angiogenesis is vital to tumor growth (16). In this study, the MTT assay was used to detect the effect of different concentrations of HDAC inhibitors on the proliferation and apoptosis of breast cancer-resistant cells. Flow cytometry was used to detect cell cycle changes, as well as western blotting to assess the protein level in cancer cells.

Our study showed that HDAC inhibitors significantly decrease the proliferation, invasion, and migration of breast cancer cells. We also observed that treatment of cancer cells by HDAC inhibitor arrests the cell cycle in G2/M phases. Furthermore, our results revealed that HDAC inhibitor suppresses the invasion and migration of cancer cells. In contrast, the proportion of S-phase cells increased significantly after treatment. However, the results revealed that the anti-cancer potential of HDAC inhibitor significantly improved in combination with PI3K inhibitor. This evidence indicated that the anti-cancer potential of HDAC inhibitors could be promoted in combination with PI3K inhibitors against tamoxifen-resistant breast cancer cells through the direction of the cancer cells to the S phase. 

High rates of mortality in patients with various cancers are due to the emergence of metastatic cells and their invasion of distant tissues (17). The adhesion between cells plays an important role in the migration of cells and is the key link between invasion and tumor deterioration (18, 19). In this study, a significant decrease was observed in the invasion and migration ability of the breast cancer cells after treatment by HDAC inhibitor and PI3K inhibitor. It showed that HDAC inhibitors promote the binding of tamoxifen to G protein-coupled estrogen receptor (GPR30), as well as activating Src protein (a proto-oncogene tyrosine-protein), enhancing Calpain activity (calcium-activated protease), regulating kinase cascade, stimulating expression of focal adhesion kinase, and as a result, inhibiting cell invasion and metastasis (20). To further identify the effects of HDAC inhibitor and PI3K inhibitor on the invasion and migration of the cancer cells, we evaluated their effect on metastasis-related protein levels, Vimentin, and E-cadherin. The results demonstrated that HDAC inhibitor and PI3K inhibitor significantly increased levels of E-cadherin as well as significantly decreasing levels of Vimentin in the cancer cells. These results reveal that HDAC inhibitor and PI3K inhibitor inhibit invasion and migration of the breast cancer cells through regulation of E-cadherin and Vimentin as metastasis-related proteins.

PI3k/Akt/mTOR signal pathway is an important signal transduction pathway that regulates cell growth, which is vital in tumor occurrence, development, invasion, migration, and therapeutic resistance (21). PI3k is a phosphatidylinositol kinase that can catalyze phosphatidylinositol 4,5-bisphosphate to phosphatidylinositol 3,4,5-triphosphate. On the other hand, up-regulation of phosphatidylinositol 3,4,5-triphosphate stimulates Akt activation, thereby promoting a series of biological processes (22). In addition, mTOR is a downstream molecule of this signaling pathway that can positively phosphorylate Akt, which further activates the PI3k/Akt/mTOR signaling pathway (23). In this study, we found that HDAC inhibitors can significantly inhibit p-PI3k, p-Akt, and p-mTOR; while PI3k inhibitor was used to inhibit the activity of the PI3k/Akt/mTOR signaling pathway. Our results demonstrate that HDAC inhibitor and PI3K inhibitor can overcome breast cancer resistance to tamoxifen through down-regulation of the PI3k/Akt/mTOR signaling pathway.

## Conclusion

In general, we showed that HDAC inhibitor and PI3k inhibitor inhibit the migration, invasion, and proliferation of tamoxifen-resistant breast cancer cells and may be useful for the treatment of patients with breast cancer. Our study suggested that HDAC inhibitor and PI3k inhibitor decrease the proliferation of tamoxifen-resistant breast cancer through inhibition of the PI3k/Akt/mTOR signaling pathway. However, further studies are required to identify other anti-cancer mechanisms of HDAC inhibitors and PI3k inhibitors in order to better manage patients with breast cancer.

## Authors’ Contributions

L W and C G designed the experiments; L W and Y X performed experiments and collected data; C G discussed the results and strategy; C G supervised, directed, and managed the study; L W, Y X, and C G approved the final version of the manuscript.

## Conflicts of Interest

The authors declare no conflicts of interest.
